# Predicting drug activity against cancer cells by random forest models based on minimal genomic information and chemical properties

**DOI:** 10.1371/journal.pone.0219774

**Published:** 2019-07-11

**Authors:** Alex P. Lind, Peter C. Anderson

**Affiliations:** Physical Sciences Division, University of Washington Bothell, Bothell, Washington, United States of America; Liverpool John Moores University, UNITED KINGDOM

## Abstract

A key goal of precision medicine is predicting the best drug therapy for a specific patient from genomic information. In oncology, cancers that appear similar pathologically can vary greatly in how they respond to the same drug. Fortunately, data from high-throughput screening programs often reveal important relationships between genomic variability of cancer cells and their response to drugs. Nevertheless, many current computational methods to predict compound activity against cancer cells require large quantities of genomic, epigenomic, and additional cellular data to develop and to apply. Here we integrate recent screening data and machine learning to train classification models that predict the activity/inactivity of compounds against cancer cells based on the mutational status of only 145 oncogenes and a set of compound structural descriptors. Using IC_50_ values of 1 μM as activity cutoffs, our predictive models have sensitivities of 87%, specificities of 87%, and yield an area under the receiver operating characteristic curve equal to 0.94. We also develop regression models to predict log(IC_50_) values of compounds for cancer cells; the models achieve a Pearson correlation coefficient of 0.86 for cross-validation and up to 0.65–0.73 against blind test sets. Predictive performance remains strong when as few as 50 oncogenes are included. Finally, even when 40% of experimental IC_50_ values are missing from screening data, they can be imputed with sufficient reliability that classification accuracy is not diminished. The presented models are fast to generate and may serve as easily implemented screening tools for personalized oncology medicine, drug repurposing, and drug discovery.

## Introduction

A fundamental goal of precision medicine is to link genetic variability with clinical-pathological indices to predict whether disease in a specific patient will respond to a specific treatment [1–3]. Recent advances in sequencing techniques and broad-scale biologic databases have rapidly increased the amount of available disease-relevant information that can be used to tailor therapy to the complex genomic context of the individual patient [4,5].

One area where precision medicine is of particular interest is cancer treatment. Cancers that appear similar pathologically often respond differently to the same drugs, complicating therapy [6–8]. The applicability of precision medicine to oncology is highlighted by the fact that patient-specific targeted therapy has already been implemented and is being developed for an increasing number of cancers [8–12]. Large libraries of drugs and experimental compounds have been screened against numerous cancer cell lines featuring heterogeneous genomic profiles [13], and recent studies have shown that high-throughput screening can identify novel molecular genomic determinants of drug sensitivity [13–19]. Data sets generated by such screening studies thus serve as crucial starting points for matching effective therapeutics with specific cancers based on genomic profiles of cancer cells. For example, the Genomics of Drug Sensitivity in Cancer (GDSC) project [20] data set contains experimental activity data for > 200,000 drug-cancer cell combinations. Accordingly, several recent studies have used the GDSC data set to train and test computational models that predict anti-cancer activities of drugs [21–23]. Despite the growing body of available genomic data, however, methods to better match patients to drugs remain in high demand.

A bottleneck in exploiting screening data for personalized medicine is generating accurate computational models that link genomic profiles to drug response [4]. Several factors can contribute to complicating this task. For example, the high-dimensionality of screening data (when the number of reported gene-drug or gene-cell type combinations greatly exceeds the number of samples) increases the chance of false positive associations [24]. A second complicating factor is that relying exclusively on one specific type of genomic information, such as gene mutation status, may have limitations, as many cancer gene mutations are merely passengers and not drivers (i.e. not mutations that give a fitness advantage to the cells that carry them) of cancer. The driver role of genes is frequently revealed only by information other than mutation status, including epigenomic, copy number variation, and gene expression data [24]. An additional complicating factor is that many machine learning methods generally have greater predictive power when trained with larger numbers of relevant descriptors (e.g. greater volume of genomic information), yet it can be costly and time-consuming to experimentally obtain a larger amount of genomic information for cell samples in a clinical setting. Accordingly, it would be helpful if balance could be achieved between model accuracy and the complexity and scale of data required by the model. It would be particularly beneficial to have accurate predictive models that required only a small amount of genomic data as experimental input.

Numerous computational methods for predicting cancer cell susceptibility to drugs have already been developed [4,21–23]. Most of these methods involve machine learning algorithms, including kernel-based methods, such as support vector machines and Bayesian efficient multiple kernel learning (BEMKL) models, and feature selection-based methods, such as random forests, elastic nets, neural networks, and more recently introduced deep-learning approaches [25,26]. However, many of these methods require a large volume of genomic, epigenomic, and/or additional types of cellular data to train and to apply to test samples, rely on prior information about the mode of action of drugs, such as their protein targets or biological pathways, or are sufficiently complex to be beyond the ability of many clinicians and non-computational researchers to apply. Moreover, many methods are tailored to specific cancer types, including breast cancer [4] and leukemia [24]. Given that more rare cancer types are less likely to have prediction methods devoted to them, a generalized prediction tool applicable to multiple cancer types would be valuable. Finally, most previous computational studies [21,23] that are based on the frequently applied GDSC data set involve earlier releases of the data set that contain ~140 drugs and ~700 cancer cell lines, thus exploring narrower ranges of chemical space and cell lines than are available in more recent releases. For these reasons, there remains a need for robust models that (i) require a minimal amount of genomic data for a given cell type, (ii) are generalizable across numerous cancer cell types, (iii) are trained and tested on as wide a range of drugs and cell lines as are currently available, and (iv) whose methodology is simple enough to be employed by clinicians and researchers in non-computational specializations.

To address this need, we have generated accurate machine learning models that predict activities of small-molecule drugs against cancer cells using a limited quantity of genomic mutation data as the only required experimentally derived input. Although relying on mutation data alone may have limitations in some cases, we show that models that rely on such data can nonetheless achieve high accuracy, provided that (i) the training set contains a sufficiently large number of drug-cell line combinations and (ii) the training set is augmented by chemical descriptors of the drugs’ structures. We selected random forests from the wide range of available machine learning methods because they rank among the most accurate methods, run efficiently on large data sets, can handle large numbers of input variables without variable deletion, estimate important features for classification, are simple to implement, are relatively insensitive to noise and outliers, are nonparametric, and can effectively impute missing data [27–29]. Moreover, random forests have ranked among the top-performing prediction algorithms in the NCI-DREAM drug sensitivity prediction challenge [4,30], and they been applied successfully in several other drug sensitivity studies [31–33].

Oncogenes are genes involved in regulating cell growth that can cause cells to grow continuously to form a tumor if they become defective. The mutational status of oncogenes in cancer cells can often predict how cancer cells will respond to specific drugs [11,34–37]. Accordingly, we applied random forest machine learning to predict the activities of 225 approved and experimental compounds against 990 cancer cell lines based on the mutational status of only 50 cellular oncogenes and ~1200 chemical descriptors. First, we used experimentally measured IC_50_ values (the half-maximal inhibitory concentration of a compound with respect to cell viability) to train random forest classification models that predict compound activity (active vs inactive) against cancer cells irrespective of cancer cell line. The models have high sensitivity and specificity, yielding an area under the receiver operating characteristic curve equal to 0.94. Second, we show that up to 40% of experimental IC_50_ values can be imputed, if they are missing, prior to model training without decreasing model accuracy. The ability to accurately impute IC_50_ values is useful in the common situation where compound activity values are missing from experimental data sets. Third, we trained random forest regression models that predict log(IC_50_) values based on the same set of descriptors as those used for the classification models. These regression models achieve a Pearson correlation coefficient equal to 0.86 and a Spearman rank correlation of 0.83 in 5-fold cross-validation and Pearson and Spearman rank correlations of ~0.7 in blind tests against new compounds. Simple to train and apply, the presented models may serve as useful *in silico* tools in drug discovery, drug repurposing, and personalized oncology medicine.

## Materials and methods

### Experimental activity data set

Experimental data for cancer cell drug sensitivity were obtained from the 2016 release of the Genomics of Drug Sensitivity in Cancer (GDSC) project [20]. This data set contains 1001 cancer cell lines and 225 drugs (S1 Fig), including experimental and approved anticancer drugs. Each cell line is described by a set of genomic features pertaining to 19,100 genes, such as mutation and methylation status and copy number variation. For most of the drug-cell line combinations, the experimentally measured log(IC_50_) is reported, where IC_50_ is the drug concentration required to eradicate 50% of the cells in the cell line. We removed from the data set all cell lines lacking mutation data for at least 20 genes and all drug-cell line combinations for which no IC_50_ values are reported. There remained a total of 990 cell lines (S2 Fig) and 180,000 drug-cell line combinations with measured IC_50_ values. IC_50_ values range from 5x10^−11^ M (the most sensitive drug-cell combination) to 0.4 M (the least sensitive drug-cell combination).

### Generating oncogene mutation profiles for cancer cell lines

Of the 19,100 genes in the GDSC experimental activity data set, 145 oncogenes were selected (S1 Table), and all other genes were removed from the data set. The 145 selected oncogenes are those that have the greatest information entropies, that is, the oncogenes for which the number of cell lines having mutations is closest to the number of cell lines lacking mutations across the 990 cell lines. For each cancer cell line, a 145-element vector describing its oncogene mutational spectrum was generated. Oncogenes possessing any type of mutation (sequence variation) were assigned a value of 1; oncogenes lacking mutations were assigned a value of 0.

### Calculating chemical-descriptor fingerprints for drug molecules

The SMILES structures of the 225 drugs in the activity data set were retrieved directly from the data set. The CheS-Mapper [38] application was used to generate a set of chemical descriptors for each drug based on the drug’s two-dimensional structure. The descriptor set included 192 Chemistry Development Kit [39] (CDK) descriptors and 1024 Extended Connectivity Fingerprints [40] (ECFP6) descriptors, yielding a fingerprint containing 1216 chemical descriptors.

### Estimating oncogenes whose mutation statuses have highest predictive value for cancer cell sensitivity to drugs

Out of the set of 145 oncogenes that we selected to describe the mutation profiles of cancer cell lines, we sought to identify the subset of oncogenes whose mutation status is most highly predictive of cell sensitivity to anticancer drugs. We focused on oncogenes because many oncogenic mutations have been shown to effectively discriminate between cells that are responsive and unresponsive to chemotherapeutic agents [11,34–37]. For each of the 180,000 drug-cell line combinations, the 145-element oncogene mutation vector for the cell line was joined with the 1216-descriptor structural fingerprint of the drug, yielding a final vector containing 1361 total elements that combines cellular and chemical information. The vectors were combined to yield a 180,000x1362 data matrix, in which the first column contains the activity class of the drug. An IC_50_ of 1 μM was initially selected as the cutoff for active drugs, as 1 μM is a commonly used threshold for distinguishing activity vs inactivity in drug screening campaigns. If IC_50_ ≤ 1 μM, the drug was designated as *active* against the cell line; otherwise, the drug was designated as *inactive*. We applied the stand-alone C++ random forest program Ranger [41] to the data matrix in order to construct a random forest binary classification model for predicting drug activity class, using the oncogene mutation status and chemical-descriptor fingerprint columns as descriptors. Five hundred trees were used, and a default *m*_try_ value of 37 was applied. The relative importance of each oncogene’s mutation status for activity prediction was measured by its computed Gini impurity index [42], where a greater index corresponds to greater relative importance for prediction. The oncogenes were ranked in order of decreasing Gini impurity index.

In addition to estimating the relative importance of each oncogene mutation status for the complete set of drugs, we also calculated the relative importance of oncogene mutation status for each drug individually. For each drug, a random forest model was trained using only the subset of the full data matrix containing the drug, and the Gini impurity index was computed for each oncogene mutation status.

### Training and validating random forest classification models to predict drug activity

We applied random forest classification modeling to predict activity vs inactivity of drugs against cancer cell lines based on a combination of mutation profiles of the 145 cellular oncogenes and chemical fingerprints of drugs using the 180,000x1362 data matrix described above. An IC_50_ of 1 μM was initially selected as the cutoff for active vs inactive drugs.

We generated a random forest classification model using 5-fold cross-validation. The first 80% (144,000) of the rows of the full matrix were selected as a training set, while the remaining 20% (36,000) of the rows were reserved as a test set. The Ranger program was applied to the training data set in order to train a model for predicting drug activity status, using 500 trees and a default *m*_try_ value of 37. The trained model was subsequently used to predict the activities of the 36,000 drug-cell line combinations in the test set. We assessed the performance of the model by computing the overall accuracy, sensitivity, specificity, false positive rate (FPR), negative predictive value (NPV), and Cohen’s kappa statistic [43] (*κ*), and negative predictive value (NPV), which are given by

$$accuracy=\frac{TP+TN}{TP+TN+FP+FN}$$ (1)

$$sensitivity=\frac{TP}{TP+FN}$$ (2)

$$specificity=\frac{TN}{TN+FP}$$ (3)

$$FPR=\frac{FP}{TN+FP}$$ (4)

$$NPV=\frac{TN}{TN+FN}$$ (5)

$$k=1‐\frac{1‐p_{0}}{1‐p_{e}}$$ (6)

where TP, TN, FP, and FN are the numbers of true positives, true negatives, false positives, and false negatives, respectively, and *p*_0_ is the observed accuracy calculated in Eq 1. Given a total number of instances *N*, the parameter *p*_e_ is the hypothetical probability of chance agreement, which is calculated as

$$p_{e}=\frac{\left(TP+FN)(TP+FP\right)}{N^{2}}+\frac{\left(FP+TN)(FN+TN\right)}{N^{2}}$$ (7)

This process was repeated such that each block of 20% of the data set rows had a turn being reserved as the test set. Model performance was assessed for each round, and mean values for accuracy, sensitivity, specificity, false positive rate, and kappa statistic for all five rounds were calculated.

To further validate the classification models, we applied a stricter version of cross-validation in which we ensured that the training set and test set never contain any drugs in common (‘blind’ testing). In the cross-validation scheme described previously, it is possible for a given drug *D* to be found in both the training and test sets. For the stricter cross-validation, however, every drug-cell line combination involving drug *D* occurs exclusively in the training set or in the test set. For each of 20 rounds of cross-validation, we randomly selected 10 (out of 225) drugs to withhold from the training set, leaving 215 drugs in the training set with which a new random forest classification model was trained. The activities of the 10 withheld drugs were then predicted using the new model. This scheme allowed us to simulate a scenario where the active/inactive class needs to be predicted for a new drug that has not been involved in any prior model training. The strict cross-validation was performed using an IC_50_ activity cutoff of 1 μM.

As a final validation step, we applied *y*-randomization, a method to control for the possibility that strong model performance is attributable to chance correlation between descriptors [44]. After the training and test sets were generated, the class labels (*active* and *inactive*) in the training set were randomly shuffled, and the model computed from the shuffled training set was tested on the non-shuffled test set. This process was repeated five times, and statistical metrics for model performance were computed for each iteration.

We further assessed the performance of binary classification models over a wide range of IC_50_ cutoff values. The method described above was repeated using each of the following cutoff values (in μM): 0.01, 0.05, 0.08, 0.1, 0.5, 0.6, 0.7, 0.8, 2, 3, 4, 5, 6, 7, 8, 9, 10, 12, 14, 16, 18, 20, 30, 40, 50, 75, 100, 150, 200, 250, 300, 350, 400, 450, 500, 600, 700, 800, 900, 1000, 2500, 3000, 4000, 5000. The extreme cutoff values lead to large class imbalance, in which one class (*active* or *inactive*) is significantly more numerous than the other class. Large class imbalance can impair the performance of random forest and other machine learning models, especially for predicting the minority class [45]. To address the issue of large class imbalance, for all cutoff values at which the minority class constitutes <20% of the total instances, we applied the synthetic minority over-sampling technique (SMOTE) [46] using five nearest neighbors during sampling, as implemented using the *smotefamily* package in the R statistical software (http://www.R-project.org/). SMOTE achieves a more balanced data set by creating synthetic minority class examples in order to over-sample the minority class and by under-sampling the majority class. The balanced data sets were subsequently used for building random forest models applying 5-fold cross-validation and 500 trees.

To quantify the overall performance of the classification models, a receiver operating characteristic curve was generated from the complete set of false positive rates and sensitivities computed across all the IC_50_ cutoffs. The area under the curve was calculated using the *DescTools* package in R.

### Finding minimum set of oncogene mutations for drug activity prediction

We hypothesized that a set of oncogenes whose mutation status is least important for accurately classifying drug activity could be omitted from the data matrix while still allowing strong model performance. To test this hypothesis, we selected the *N* (*N* = 5, 10, 15, …, 100) oncogenes whose mutation status had the greatest Gini impurity indices (determined as described previously) and retained only those oncogenes and the 1216 chemical fingerprints in the matrix. For each value of *N*, random forest models were generated from the reduced matrix using 5-fold cross-validation with 500 trees, and the same set of IC_50_ cutoff values as listed previously were utilized. The area under the receiver operating characteristic curve was calculated for each *N* value.

### Imputing missing experimental IC_50_ values

To simulate the effects of missing experimental IC_50_ values in the raw data set, we randomly removed 10%, 20%, 30%, and 40% of the IC_50_ values from the 180,000x1362 data matrix. The missing values were subsequently imputed using four different methods implemented in R software packages: (i) *missForest*, which imputes values using random forests (applying 500 trees and 10 iterations); (ii) *k*-nearest neighbors (*k* = 9) using the *impute* package; (iii) logistic regression with lasso [47] (‘lassoC’) using the *imputeR* package with 100 maximum iterations; and (iv) recursive partitioning with regression trees [48] (‘rpartC’) using the *imputeR* package with 100 maximum iterations. The imputed IC_50_ values were compared to the true values. Random forest classification models were subsequently trained by 5-fold cross-validation using 80% of the instances in the imputed-data matrices as training data. IC_50_ cutoffs of 1 μM were applied for separating active from inactive compounds. Model performance statistics were calculated using test sets taken from the original (non-imputed) data matrix. This process was repeated for the matrices containing reduced numbers of oncogene mutation descriptors described in the previous section.

### Training and validating random forest regression models to predict drug activity

We trained random forest regression models to predict log(IC_50_) values directly using the data matrix containing all drug-cell line combinations but only the 50 most important oncogene mutation statuses and the 1216 chemical descriptors. Random forests used 500 trees, and 5-fold cross-validation was applied. Pearson correlation coefficients and Spearman rank correlation coefficients were computed for the predicted and actual values of log(IC_50_). Further validation was performed using *y*-randomization, in which the original log(IC_50_) values of the training sets were randomly shuffled prior to model training. Similarly to our classification model validation process, we also performed stricter leave-drug-out cross-validation on the regression models. A hold-out drug was randomly selected, all records involving the drug were removed from the full data set, and a regression model was trained on the data set containing the remaining 224 drugs and 990 cell lines. Pearson correlation coefficients and Spearman rank correlations were subsequently computed for the predicted and actual values of log(IC_50_) for the eliminated drug. This process was repeated for a total of nine separate randomly selected drugs.

### Assessing baseline performance of classification and regression random forest models using dataset-based methods

To estimate the baseline performance of our classification random forest models, we applied several dummy classifiers, including the zero rule algorithm [49], stratified prediction, uniform random prediction, and the *k*-nearest neighbors algorithm. Each baseline assessment was performed with 10-fold cross-validation. For the zero rule algorithm baseline estimation, the majority compound activity class (*inactive*) was assigned to every instance of the test set predictions. For the stratified baseline estimation, the *active*-vs-*inactive* distribution among the test set predictions was set equal to the *active*-vs-*inactive* distribution among the training set, and predictions were randomly assigned to the test set following this distribution. For the uniform random baseline estimation, *active* vs *inactive* classes for the test set were predicted at random with equal probability. In the *k*-nearest neighbors algorithm, the prediction for each instance of the test set was set equal to the majority class of the 9 nearest neighbors in the training set (*k* = 9). Classification performance was assessed for each baseline method by the metrics of overall accuracy, negative predictive value, and kappa statistic, calculated at IC_50_ cutoff values of 0.1 μM, 1 μM, and 10 μM.

Similarly, to estimate the baseline performance of the regression random forest models, we applied three dummy regressors, including the zero rule algorithm, quantile prediction [50], and the *k*-nearest neighbors algorithm (*k* = 9). For the zero rule algorithm baseline estimation, the mean log(IC_50_) from the complete data set was assigned to every instance of the predictions. For the quantile prediction method, each test set prediction was assigned as a specified quantile of the log(IC_50_) distribution of the training set, with the specified quantiles ranging from 5% to 95% at increments of 5%. In the *k*-nearest neighbors algorithm, the predicted log(IC_50_) for each instance of the test set was set equal to the average log(IC_50_) of the 9 nearest neighbors in the training set. Regression performance was evaluated by the root-mean-square error (RMSE).

### Assessing baseline performance of classification and regression random forest models by comparison to other machine learning methods

We also sought to establish a method-based baseline against which to assess the classification and regression performance of random forest models on the GDSC data set. Accordingly, we used the same data set to classify drug activity/inactivity with IC_50_ cutoffs of 0.1 μM, 1 μM, and 10 μM and to predict log(IC_50_) values by regression, applying a few commonly used machine learning algorithms, including support vector machine (SVM), single-layer artificial neural network, and multi-layer deep-learning neural network.

The SVM classification and regression models were trained using the sofia-ml suite of algorithms (https://code.google.com/archive/p/sofia-ml/). The single-layer neural network and deep-learning network classification and regression models were trained using the R interface to the scalable, open-source H2O machine learning platform (https://cran.r-project.org/web/packages/h2o/index.html). For the single-layer neural network, 900 neurons were used in the hidden layer, as this value is ~2/3 the total number of descriptors (1362) used in the data set. For the deep-learning network, two hidden layers were applied, each of which likewise contained 900 neurons. In each neural network, tanh was used as the activation function and 1 million iterations were performed. A stochastic gradient descent learner type and a stochastic loop type were applied in conjunction with a regularization parameter (lambda) equal to 0.1.

### Data set and script availability

The 180,000x1362 GDSC data set used in this study and execution and analysis scripts are available at the protocols.io repository: dx.doi.org/10.17504/protocols.io.3j9gkr6

## Results

### Most important oncogene mutations for predicting cancer cell sensitivity to drugs

**Fig 1:**
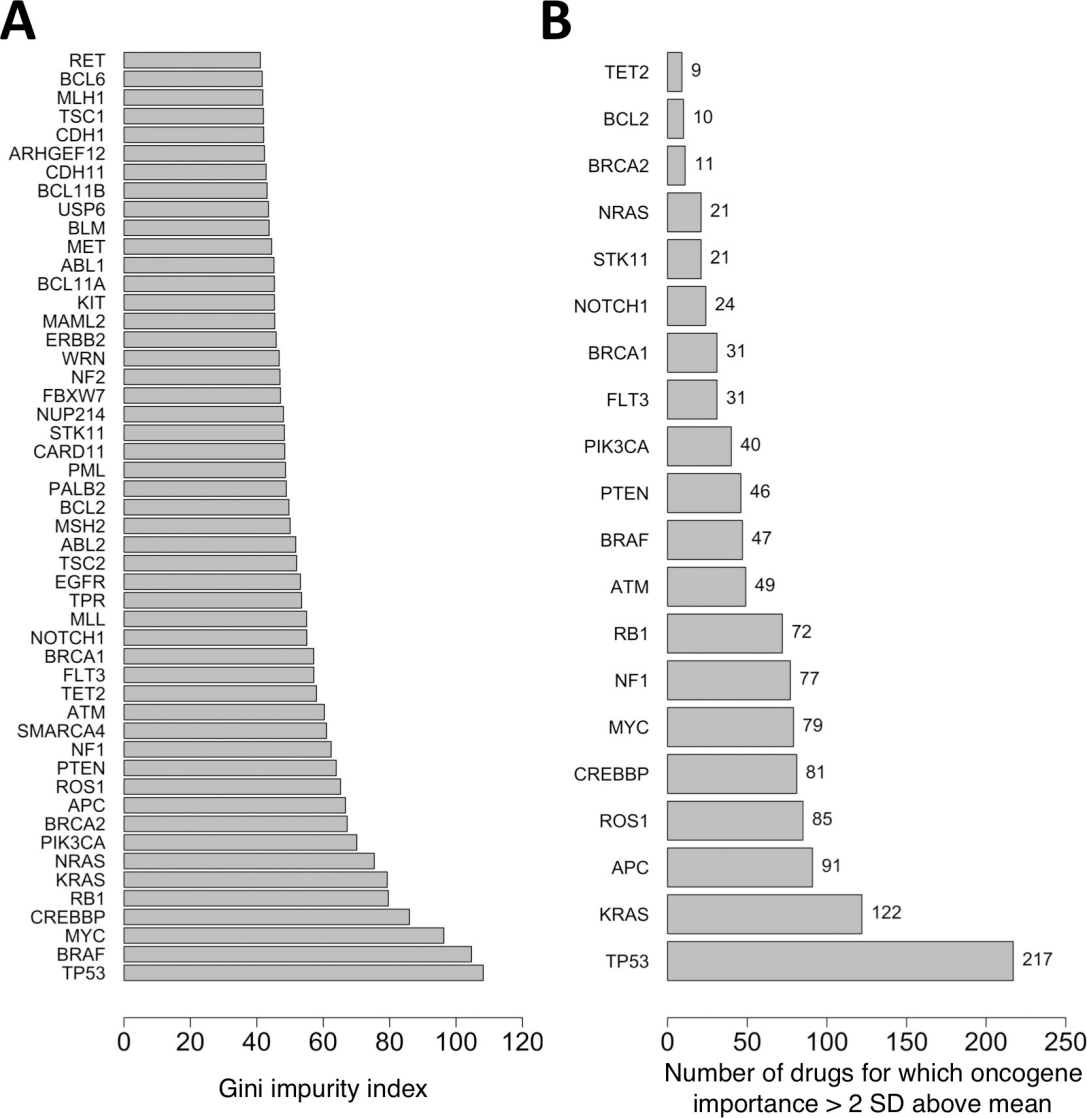
Relative importance of mutation statuses of oncogenes for predicting drug activity against cancer cell lines. (A) Gini impurity indices calculated for entire data set consisting of 225 drugs and 990 cancer cell lines. The 50 most important oncogenes are shown. (B) The top-ranking oncogenes for each individual drug were computed as those whose relative importance is >2 standard deviations above the average oncogene importance for that drug. The 20 oncogenes that are top-ranking for the greatest number of drugs are shown. Gini impurity indices for the mutation statuses were computed from random forest classification models at an IC_50_ activity cutoff of 1 μM.

The mutational statuses of the 145 analyzed oncogenes from the GDSC data set have a wide range of relative importance for predicting cancer cell sensitivity to anticancer drugs (Fig 1). Here, the sensitivity of a cancer cell line to a drug was evaluated according to whether the drug is active against the cell line (IC_50_ ≤ user-specified cutoff). An activity cutoff IC_50_ of 1 μM was applied to separate active from inactive compounds. We estimated the relative importance of each oncogene’s mutational status for correctly classifying a drug as active or inactive against the cell lines by calculating its Gini impurity index during random forest generation.

For the complete data set including all 225 drugs and 990 cancer cell lines in the GDSC data set, the mean Gini impurity index and standard deviation among all 145 oncogenes are 36 and 18, respectively (Fig 1A). The four oncogenes whose mutational statuses are the most important variables for predicting cell line sensitivity to the 225 drugs collectively are *TP53*, *BRAF*, *MYC*, and *CREBBP*, which have Gini impurity indices of 108, 105, 96, and 86, respectively.

For predicting sensitivity of the 990 cancer cell lines to each drug individually, *TP53* likewise ranks as the most important oncogene (Fig 1B). For each of the individual 225 drugs in the GDSC data set, we generated a separate random forest model to predict its activity against the cell lines. All oncogene mutation statuses whose Gini impurity indices are greater than two standard deviations above the mean Gini impurity index for the drug were designated as top-ranking oncogenes. *TP53* is top-ranking for 217 out of the 225 drugs, followed by *KRAS*, *APC*, *ROS1*, *CREBBP*, and *MYC*, which are top-ranking for 122, 91, 85, 81 and 79 drugs, respectively.

These findings are consistent with known associations between *TP53* and *MYC* mutation status and drug sensitivity [14,51], as well as associations between *BRAF*-mutated cell lines and sensitivity to several types of anticancer drugs, including MEK1/2 inhibitors [14,52].

### Predicting drug activity vs inactivity against cancer cell lines based on mutational status of 145 oncogenes and chemical descriptors of drugs

We trained random forest binary classification models that predict the activity/inactivity class of anticancer drugs against cancer cell lines using as input the mutational status of 145 cellular oncogenes and 1216 drug chemical descriptors. Models were trained using 5-fold cross-validation, where the test set of each fold was withheld from training in order to measure the predictive power of the model. We initially selected 0.1 μM, 1 μM, and 10 μM as IC_50_ cutoff values that distinguish active from inactive compounds, such that the compound is considered active against the cell line if its IC_50_ ≤ cutoff. At these cutoff values, all of the models have strong performance statistics, achieving >80% accuracy, sensitivity, and specificity and Cohen’s kappa statistic (*κ*) >0.60 (Table 1). As shown in Table 1, at IC_50_ cutoff values of 0.1 μM, 1 μM and 10 μM, the values of Cohen’s kappa statistic for the random forest classification models are greater than their respective baseline values of 0.67, 0.68 and 0.60 yielded by the *k*-nearest neighbors algorithm (*k* = 9). The kappa statistic gauges overall prediction strength, including the tradeoff between specificity and sensitivity, in a single metric. These greater values of the kappa statistic indicate that the random forest models offer a better balance between sensitivity and specificity than baseline, particularly at lower IC_50_ cutoffs.

**Table 1:** Performance metrics for random forest binary classification models. Models were trained at IC_50_ cutoff values of 0.1 μM, 1 μM, and 10 μM, using 145 cellular oncogene mutation statuses among the set of predictors. Reported errors are calculated as standard deviations from 5-fold cross-validation. Baseline values of accuracy, negative predictive value, and Cohen’s kappa statistic at each cutoff value are shown in parentheses. The kappa statistic gauges overall prediction strength, including the tradeoff between specificity and sensitivity, in a single metric. The first baseline value within each set of parentheses is the average baseline value calculated using the tested dataset-based baseline method (dummy classifier) that leads to the highest baseline performance, as measured by Cohen’s kappa statistic; for the GDSC data set, the highest dataset-based baseline performance is yielded by the *k*-nearest neighbors algorithm (*k* = 9). The second baseline value within each set of parentheses corresponds to the overall best-performing classification machine learning method (other than random forest) that we tested for the GDSC data set, as evaluated by the kappa statistic (S2 Table). The machine learning method yielding the highest kappa statistic and overall performance other than random forest is the support vector machine.

IC_50_ Cutoff	0.1 μM	1 μM	10 μM
Accuracy (%)	93 ± 2 (93; 93)	87 ± 1 (82; 87)	82 ± 1 (80; 82)
Sensitivity (%)	88 ± 4	87 ± 2	80 ± 1
Specificity (%)	94 ± 2	87 ± 1	83 ± 1
False positive rate (%)	6 ± 2	13 ± 1	17 ± 1
Negative predictive value (%)	98 ± 1 (97; 98)	97 ± 1 (92; 97)	82 ± 1 (81; 82)
Cohen’s kappa statistic (*κ*)	0.86 ± 0.04 (0.67; 0.86)	0.74 ± 0.02 (0.68; 0.73)	0.64 ± 0.02 (0.60; 0.63)

As a further validation step beyond 5-fold cross-validation, we performed *y*-randomization. The *active* and *inactive* class labels in all the original training sets were randomly shuffled, new random forest classifier models were trained, and the new models were tested on the original test sets. The mean accuracies of the new models at IC_50_ cutoffs of 0.1 μM, 1 μM, and 10 μM fell to 62%, 49%, and 50%, respectively, from the values of >80% for the original models. Similarly, the mean values of *κ* fell to 0 at all cutoffs. This major decline in performance helps to rule out the possibility that the better performance of the original models can be attributed to chance correlations between descriptors [44].

**Fig 2:**
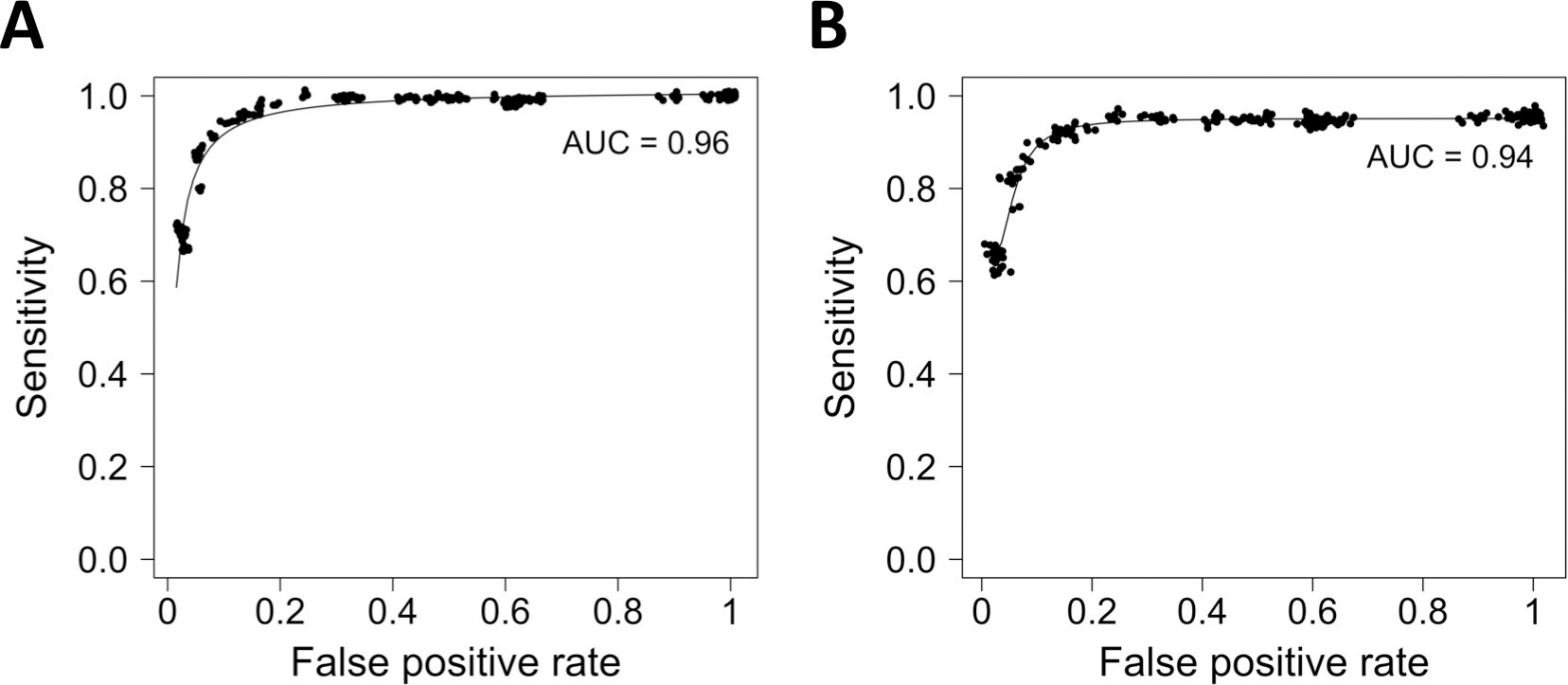
Receiver operating characteristic plots for random forest binary classification models. Random forest models were generated using (A) all 145 cellular oncogene mutation statuses and (B) the 50 most important cellular oncogene mutation statuses.

We evaluated the performance of the classifier models over a wide range of IC_50_ cutoff values between 0.01 μM and 5000 μM. The random forest classification models perform strongly overall, yielding an area of 0.96 under the receiver operating characteristic curve (Fig 2A).

In addition, we compared the classification performance of random forests with that of a few other machine learning algorithms that are frequently used in personalized medicine, including support vector machines, single-layer neural networks, and multi-layer deep neural networks (S2 Table). At the three tested IC_50_ activity cutoffs of 0.1 μM, 1 μM, and 10 μM, the support vector machine classification model has kappa values of 0.86 ± 0.04, 0.74 ± 0.02, and 0.64 ± 0.02, respectively. Interestingly, these values are identical to those of the random forest classification model (Table 1) and are considerably higher than those of the classification models generated by the single-layer neural network and the multi-layer deep-learning network, which range from as low as 0.38 to 0.60. These data indicate that for the GDSC data set, random forest classification models and support vector machine classification models perform comparably well, providing roughly equal measures of accuracy, negative predictive value, and kappa statistic at each cutoff, and perform better than the tested neural and deep-learning networks.

### Predicting activity of ‘new’ drugs that have not been seen in prior training

The random forest classification models perform strongly even when they are tested on drugs that are missing from the training sets, that is, when the trained models are ‘blind’ to the tested drugs. In order to test model performance for drugs that have not been involved in prior training, we removed randomly selected sets of 10 drugs from the full data set, leaving 215 drugs and 990 cancer cell lines. A random forest classification model was trained on the remaining data using an IC_50_ cutoff of 1 μM, and the activities of the withheld set of drugs were predicted from the model. This stricter validation process was repeated 20 times. Mean accuracy, sensitivity, and specificity for the activity predictions for the withheld drugs were 85%±7%, 79%±15%, and 84%±5%, respectively, which are only slightly lower than their corresponding values of 87% for the less strict cross-validation used previously, in which a given drug D can be present in both the training and the test sets. These performance metrics show that the classification models’ predictive performance remains strong regardless of whether the models are trained using drugs included in the test sets.

### Finding minimum set of oncogene mutations for predicting drug activity

The random forest classification models for predicting drug activity perform with roughly the same level of accuracy until only the 50 most important oncogene mutations (shown in Fig 2A) remain. We systematically eliminated from the full data matrix the least important oncogene mutation statuses (as measured previously by their Gini impurity indices) five at a time and trained a new model on each reduced data set at IC_50_ cutoffs of 1 μM and 0.1 μM, evaluating the accuracy, sensitivity, specificity, and *κ* for each resulting model. Relative to the full data matrix containing all 145 oncogenes, there is no decline in performance until fewer than 50 oncogenes remain (Table 2).

**Table 2:** Mean performance metrics for random forest binary classification models as a function of number of oncogenes in training data set. IC_50_ activity cutoff values of 1 μM and 0.1 μM were applied. Values in parentheses correspond to an IC_50_ activity cutoff of 0.1 μM.

Oncogene subset size	Accuracy (%)	Sensitivity (%)	Specificity (%)	*κ*
125 oncogenes	87 (93)	87 (88)	87 (94)	0.74 (0.86)
110 oncogenes	87 (92)	87 (87)	86 (94)	0.74 (0.84)
95 oncogenes	88 (92)	88 (88)	87 (93)	0.76 (0.84)
80 oncogenes	87 (93)	88 (88)	87 (94)	0.74 (0.86)
65 oncogenes	87 (92)	86 (87)	87 (94)	0.74 (0.84)
50 oncogenes	87 (93)	87 (88)	87 (94)	0.74 (0.86)
40 oncogenes	84 (89)	83 (88)	84 (89)	0.68 (0.78)
30 oncogenes	81 (86)	81 (86)	82 (86)	0.62 (0.72)

When fewer than the 50 most important oncogene mutation statuses remain in the data set, the accuracy, sensitivity, specificity, and *κ* metrics for the classification models begin to decline. We selected the set of 50 oncogenes as the optimum number to use in the final classification models, as this number allows the models to maintain maximal performance while minimizing the amount of required input data. The receiver operating characteristic plot for the models using the 50 top oncogenes yields an area under the curve of 0.94, showing overall performance that is almost identical to that of the original models using the full set of 145 oncogenes (Fig 2B).

### Imputing missing IC_50_ values and building classification models using imputed data

**Fig 3:**
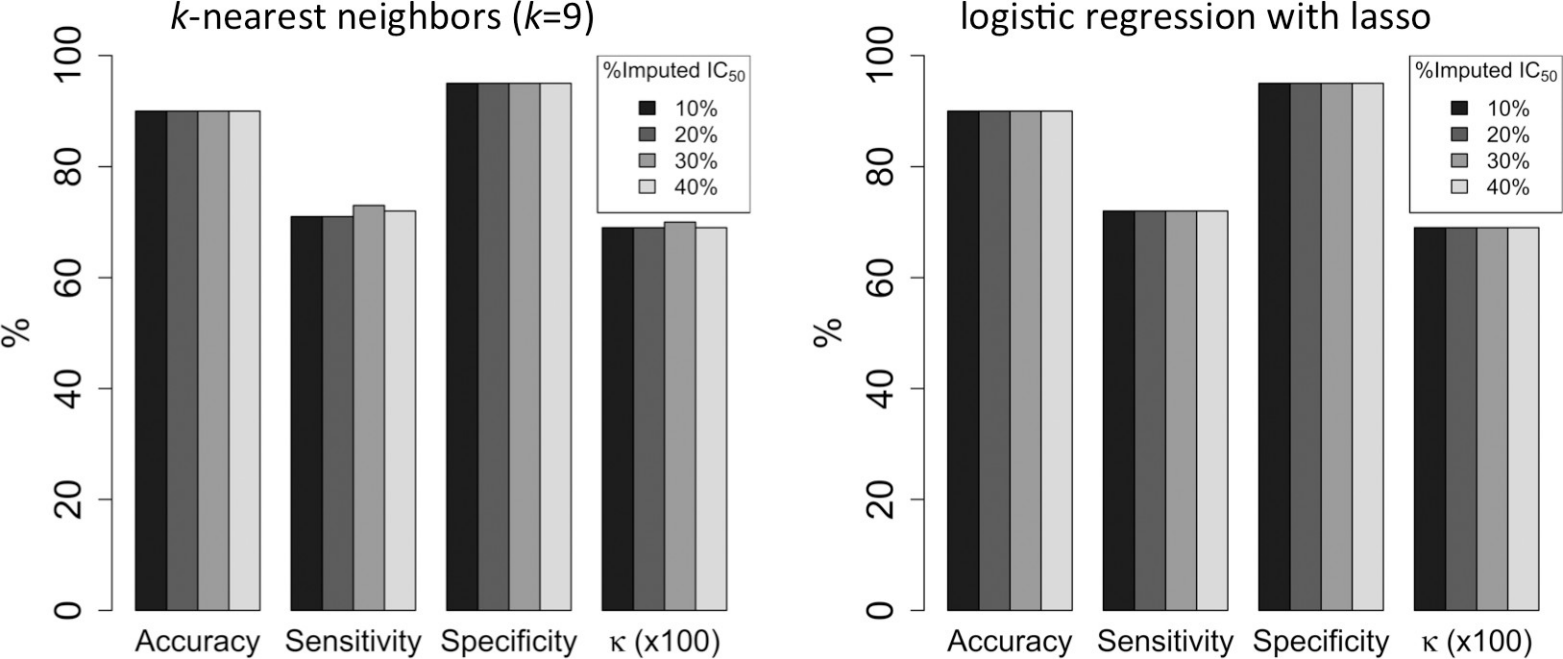
Mean performance metrics for random forest binary classification models trained on data sets containing imputed IC_50_ values. IC_50_ values were imputed by the *k*-nearest neighbors (*k* = 9) algorithm (left) and the logistic regression with lasso (‘lassoC’) algorithm (right). Percentages of imputed values range from 10% to 40%. An IC_50_ activity cutoff of 1 μM was applied. The kappa statistic (*κ*) has been multiplied by 100 for scaling.

Clinical and genomic research commonly involves missing data, and missing data can complicate and undermine the validity of research results [53]. Accordingly, we simulated the presence of missing IC_50_ data in the GDSC data set by randomly discarding 10%, 20%, 30%, and 40% of the IC_50_ values and subsequently imputing the missing values by random forest regression models. The logistic regression with lasso (‘lassoC’) and *k*-nearest neighbors (*k* = 9) algorithms impute missing IC_50_ values with sufficient accuracy that random forest classification models trained on the imputed-data sets have high mean accuracy (89%-90%), sensitivity (71%-73%), specificity (95%), and Cohen’s kappa statistic (0.69–0.70) even when up to 40% of experimental activity data are missing (Fig 3). The missForest R package performs slightly worse for the GDSC data set, yielding mean accuracies < 90% and mean kappa statistics ≤ 0.66. Moreover, the performance of missForest deteriorates when 40% of the IC_50_ values are missing, whereas the logistic regression with lasso and *k*-nearest neighbors algorithms maintain constant performance metrics across all tested percentages of missing data.

### Predicting log(IC_50_) values using random forest regression

**Fig 4:**
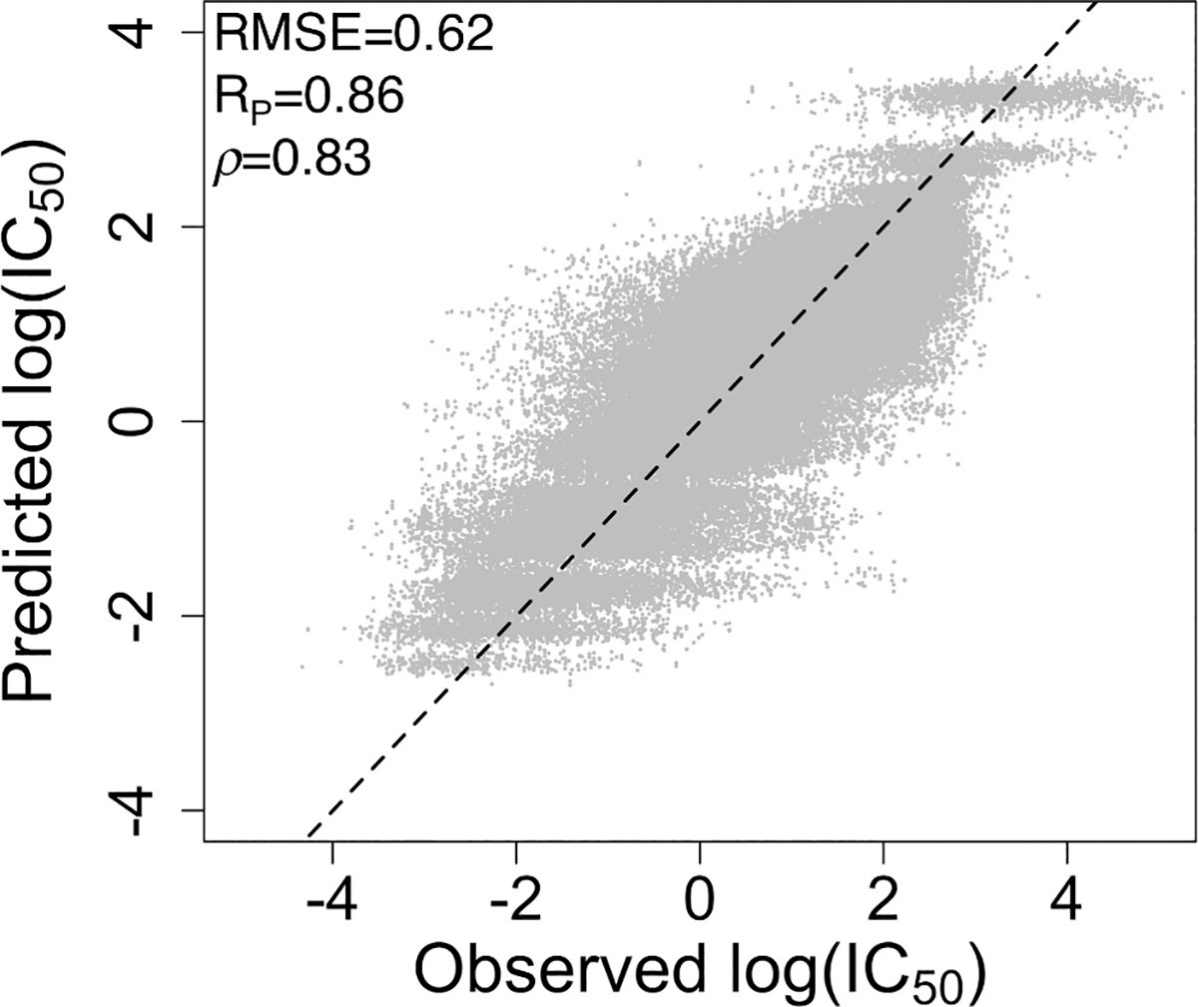
IC_50_ values for all combinations of drugs and cancer cell lines in the GDSC data set as predicted by random forest regression models. Predictions of log(IC_50_) were achieved using 5-fold cross-validation. Performance statistics are calculated for the test sets. The RMSE, Pearson correlation (R_P_), Spearman rank correlation (*ρ*), and corresponding regression line are shown.

In addition to generating classification models for predicting activity vs inactivity of drugs against cancer cell lines, we trained random forest regression models that directly predict log(IC_50_) values, applying 5-fold cross-validation such that predictions were made for all drug-cell line combinations. The models were trained using the data set containing the mutation statuses of the top 50 oncogenes and the drug chemical fingerprints. The regression models yield a root-mean-square error of 0.62 ± 0.02 log(IC_50_) unit, a Pearson correlation coefficient of 0.86, and a Spearman rank correlation of 0.83 (Fig 4). By comparison, the mean dataset-based (dummy regressor) baseline root-mean-square error of regression models is 0.89 log(IC_50_) unit for the *k*-nearest neighbors algorithm (*k* = 9) and 1.2 for both the zero rule algorithm and for the quantile prediction method.

**Fig 5:**
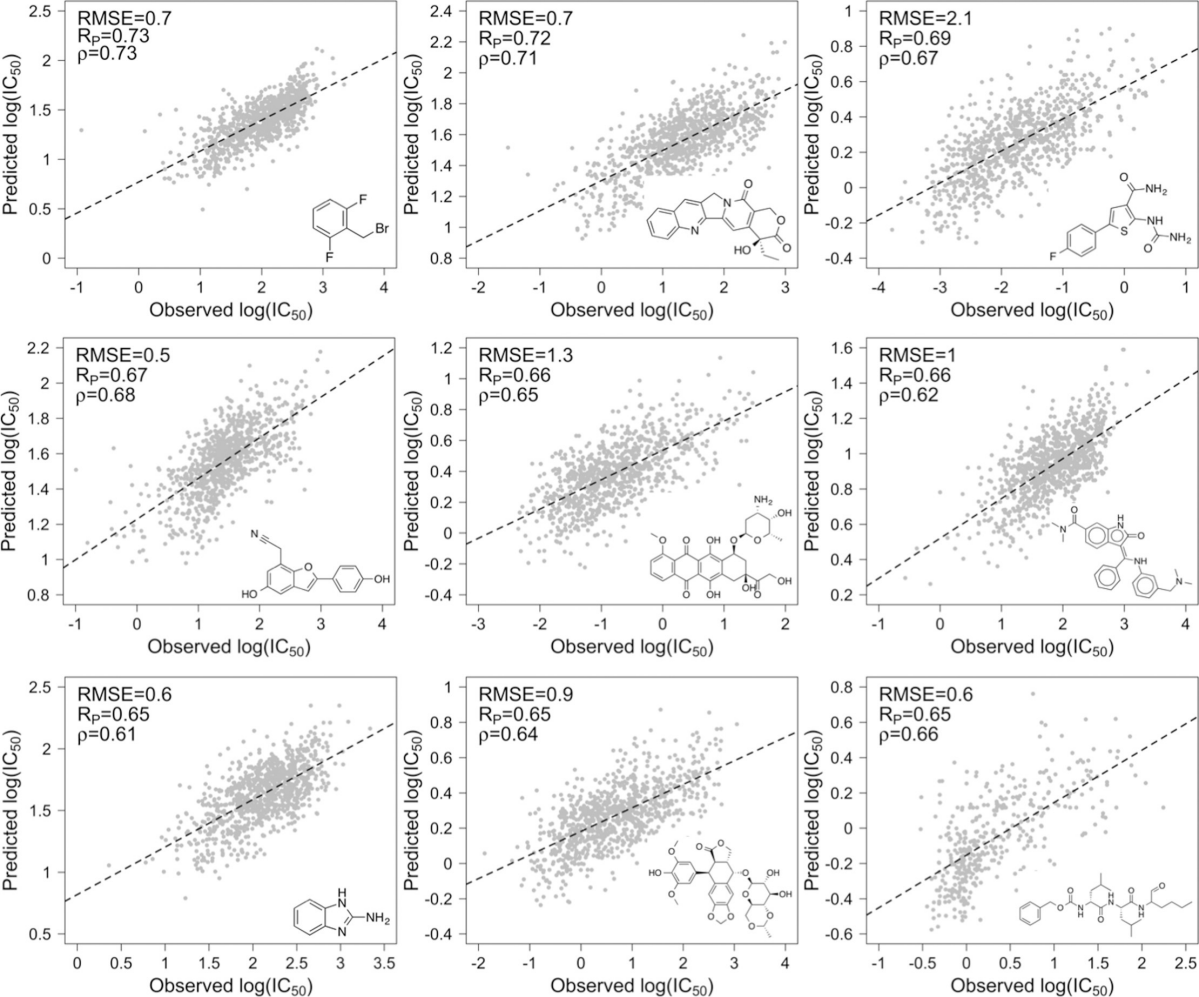
Predicted vs. observed log(IC_50_) for leave-drug-out cross-validation. Randomly selected drugs were omitted from the GDSC data set, and random forest regression models were trained using the data set containing the remaining 224 drugs and 990 cell lines. This process was performed for each of 9 drugs. The RMSE, Pearson correlation (R_P_), Spearman rank correlation (*ρ*), and corresponding regression line are shown for each omitted drug. Structures of the omitted drugs are depicted in the insets.

To perform a more rigorous test of the regression models’ predictive value, we also performed leave-drug-out cross-validation. For each of nine individual drugs that are part of the GDSC data set, a ‘blind’ random forest regression model was trained using no instances of the drug while retaining the other 224 drugs, and the predictive performance of the trained model for the omitted drug was assessed. The RMSE for the nine leave-drug-out models range from 0.6 to 2.1 log(IC_50_) units and have an average RMSE of 0.9 log(IC_50_) units (Fig 5). The Pearson correlation coefficients and Spearman rank correlations range from 0.65 to 0.73 and from 0.61 to 0.73, respectively, having averages of 0.67 and 0.66. As expected, these correlations are weaker than those for the 5-fold cross-validation, since the 5-fold cross-validation involves instances of each drug in both the training and test sets. Nevertheless, the Pearson correlation coefficients are comparable to or greater than those reported for leave-drug-out validation tests performed in several previous drug sensitivity-prediction studies applying machine learning [23,24,54–56]. The observed correlations suggest that the regression models may be applied with reasonable accuracy to predict relative activities of new compounds.

Additionally, we compared the regression performance of the random forest models with that of models generated by support vector machine, single-layer neural networks, and multi-layer deep-learning networks. The average RMSE of predicted log(IC_50_) values from 5-fold cross validation of these models are 1.10 ± 0.02, 0.72 ± 0.02, and 0.70 ± 0.01 log(IC_50_) units, respectively. For the GDSC data set, these methods thus yield higher RMSE values than does the random forest regression model, which has an RMSE of 0.62 ± 0.02 log(IC_50_) unit.

### Required computation time for random forest generation

On a desktop computer featuring two Intel 2.4 GHz processors and 12 GB of RAM, the mean wall-clock time required to train random forest classification models using the stand-alone C++ Ranger program distributed over 16 threads was 9.7 minutes. The mean wall-clock time required to train SVM classification models using the sofia-ml package was 0.1 minute, while the mean times required for the single-layer neural network and multi-layer deep-learning network using the R interface to the H2O machine learning platform were 9.5 minutes and 42.0 minutes, respectively. The mean wall-clock times required for training random forest, SVM, single-layer neural network and multi-layer deep-learning network regression models using the same programs as above were 11.8 minutes, 0.1 minute, 8.0 minutes, and 25.0 minutes, respectively.

## Discussion

An overarching goal of precision medicine is to match drugs to the specific genomic profiles of patients in order to maximize the effectiveness of treatment for the individual. In oncology, the availability of large data sets obtained from high-throughput screening campaigns against cancer cell lines has made it possible to decipher relationships between cancer cell genomic data and cellular drug sensitivity. Although many excellent computational methods have been developed to identify these relationships from experimental data, including several used in the NCI-DREAM drug sensitivity prediction challenge, they are often specialized for a small subset of cancer types, involve complex modeling techniques, and/or require large volumes of heterogeneous genomic and extra-genomic information obtained from disparate data sets. For instance, information involving RNA sequence, methylation status, copy number variation, reverse phase protein array, and biological pathway annotations is often required for maximal performance.

In an effort to create as simple a sensitivity prediction method as possible, we sought to leverage the large quantity of current publicly available screening data to create computational models that (i) are applicable to a broad range of cancer types, (ii) require only a minimal amount of experimental data to train and apply, and (iii) involve a non-parametric, well-validated machine learning technique that is simple to implement ‘out of the box’ for clinicians and researchers, including those without computational expertise.

We have shown that the activities of 225 drugs against 990 cancer cell lines can be predicted by random forests with high accuracy using only the mutation status of 50 oncogenes and ~1220 easily computed chemical descriptors of drug structures. To our knowledge, the mutation status of only 50 oncogenes is the smallest quantity of experimental data required for any recently published method. We used the GDSC data set to train binary classification random forest models that achieve overall accuracy, sensitivity and specificity >80% and an area under the ROC curve of 0.94, as well as regression random forest models that predict log(IC_50_) values with a Pearson correlation of 0.86. Interestingly, this Pearson correlation coefficient is the same as that of a previously reported regression model trained on an earlier version of the GDSC screening data set containing 608 cancer cell lines and 111 drugs [54]. Moreover, several rounds of leave-drug-out cross-validation, in which trained regression models are completely ‘blind’ to test compounds, achieve Pearson correlation coefficients and Spearman rank correlations between predicted and observed log(IC_50_) values that range from 0.65 to 0.73. The ability to predict and rank new drug activity against annotated cancer cell lines suggests that the models may serve as a useful tool in drug discovery and clinical settings when novel drugs become available but have not yet been subjected to high-throughput cell screening.

Additionally, we have demonstrated that when up to 30% of experimental IC_50_ values are missing from the GDSC data set, they can be imputed without compromising the accuracy of the classification models. This capability means that in the common scenario where the activities of a subset of drug-cell line combinations have not been experimentally measured in a data set, these combinations need not be discarded; rather, the activities can be estimated from existing data with reasonable accuracy, maximizing the size of the data set available for model training.

The overall predictive strength of our models likely stems from both the large number of drug-cell line combinations (180,000) in the GDSC data set and the broad chemical space coverage that is afforded by the 225 drugs in the data set. However, model performance likely could be improved as screening data sets continue to become larger and capture a broader range of drug chemical space and greater cancer cell line diversity. In addition, we focused exclusively on oncogene mutation status as predictive genomic cell features, but the mutation status of other types of genes may be even more highly predictive of cell sensitivity and drug activity. Finally, as demonstrated by a recent study [32], cell sensitivity prediction accuracy can be enhanced by incorporating relationships between different output responses, as implemented by multivariate random forests. In the context of the present work, incorporating relationships between drug pair sensitivities using multivariate random forests may boost the models’ predictive power.

Random forests, which we applied in the present study, offer the advantage of requiring little, if any, data preprocessing, have few parameters for the user to adjust, and compute the relative importance of individual descriptors, potentially allowing the least important descriptors not to be required as part of experimental data sets. For example, in the present study, we discarded 95 oncogene mutations from the original descriptor set of 145 oncogenes without compromising classification accuracy, minimizing the volume of required experimental data. These strengths make random forests valuable and a suitable choice for generating drug sensitivity prediction models for both clinicians and researchers.

In conclusion, we highlight the potential of the presented classification and regression models–and the methodology used to generate them–to accurately predict and rank the activities of drugs against a given cancer cell line, provided that the mutation status of at least the 50 most relevant oncogenes reported here has been determined for the cell line. These predictions may help guide drug discovery programs, assist in drug repurposing, and inform clinical decisions concerning effective drug treatments for specific cancer patients.

## Supporting information

S1 Fig Chemical structures of the 225 compounds in the GDSC data set. (TIF)

S2 Fig Distribution of cancer types among the 990 selected GDSC cancer cell lines. (TIF)

S1 Table 145 oncogenes selected from the GDSC data set as predictors of drug activity. (DOCX)

S2 Table Performance metrics for binary classification models generated by alternative machine learning algorithms. (DOCX)

## References

[1] Collins FS, Varmus H. A new initiative on precision medicine. N Engl J Med. 2015;372: 793–795. 10.1056/NEJMp1500523 25635347 PMC5101938

[2] Mirnezami R, Nicholson J, Darzi A. Preparing for precision medicine. New Engl J Med. 2012;366: 489–491. 10.1056/NEJMp1114866 22256780

[3] Friedman AA, Letai A, Fisher DE, Flaherty KT. Precision medicine for cancer with next-generation functional diagnostics. Nat Rev Cancer. 2015;15: 747–756. 10.1038/nrc4015 26536825 PMC4970460

[4] Costello JC, Heiser LM, Georgii E, Gönen M, Menden MP, Wang NJ, et al A community effort to assess and improve drug sensitivity prediction algorithms. Nat Biotechnol. 2014;32: 1202–1212. 10.1038/nbt.2877 24880487 PMC4547623

[5] Jameson LJ, Longo DL. Precision medicine–personalized, problematic, and promising. Obstet Gynecol Surv. 2015;70: 612–614. 10.1056/NEJMsb1503104 26014593

[6] Haibe-Kains B, El-Hachem N, Juul Birkbak N, Jin AC, Beck AH, Aerts H, et al Inconsistency in large pharmacogenomic studies. Nature. 2013;504: 389–393. 10.1038/nature12831 24284626 PMC4237165

[7] Li Y, Steppi A, Zhou Y, Mao F, Miller PC, He MM, et al Tumoral expression of drug and xenobiotic metabolizing enzymes in breast cancer patients of different ethnicities with implications to personalized medicine. Sci Rep. 2017;7: 4747 10.1038/s41598-017-04250-2 28684774 PMC5500564

[8] Kelloff GJ, Sigman CC. Cancer biomarkers: selecting the right drug for the right patient. Nat Rev Drug Discov. 2012;11: 201–214. 10.1038/nrd3651 22322254

[9] Slamon DJ, Leyland-Jones B, Shak S, Fuchs H, Paton V, Bajamonde A, et al Use of chemotherapy plus a monoclonal antibody against HER2 for metastatic breast cancer that overexpresses HER2. N Engl J Med. 2001;344: 783–792. 10.1056/NEJM200103153441101 11248153

[10] Chapman PB, Hauschild A, Robert C, Haanen JB, Ascierto P, Larkin J, et al Improved survival with vemurafenib in melanoma with BRAF V600E mutation. N Engl J Med. 2011;364: 2507–2516. 10.1056/NEJMoa1103782 21639808 PMC3549296

[11] La Thangue NB, Kerr DJ. Predictive biomarkers: a paradigm shift towards personalized cancer medicine. Nat Rev Clin Oncol. 2011;8: 587–596. 10.1038/nrclinonc.2011.121 21862978

[12] Tsimberidou A-M, Iskander NG, Hong DS, Wheeler JJ, Falchook GS, Fu S, et al Personalized medicine in a phase I clinical trials program: the MD Anderson Cancer Center Initiative. Clin Cancer Res. 2012;18: 1–11. 10.1158/1078-0432.CCR-12-1627 PMC4454458 22966018

[13] Sharma SV, Haber DA, Settleman J. Cell line-based platforms to evaluate the therapeutic efficacy of candidate anticancer agents. Nat Rev Cancer. 2010;10: 241–253. 10.1038/nrc2820 20300105

[14] Garnett MJ, Edelman EJ, Heidorn SJ, Greenman CD, Dastur A, Lau KW, et al Systematic identification of genomic markers of drug sensitivity in cancer cells. Nature. 2012;483: 570–575. 10.1038/nature11005 22460902 PMC3349233

[15] Barretina J, Caponigro G, Stransky N, Venkatesan K, Margolin AA, Kim S, et al The Cancer Cell Line Encyclopedia enables predictive modeling of anticancer drug sensitivity. Nature. 2012;483: 603–607. 10.1038/nature11003 22460905 PMC3320027

[16] Heiser LM, Sadanandam A, Kuo WL, Benz SC, Goldstein TC, Ng S, et al Subtype and pathway specific responses to anticancer compounds in breast cancer. Proc Natl Acad Sci USA. 2012;109: 2724–2729. 10.1073/pnas.1018854108 22003129 PMC3286973

[17] Shoemaker RH. The NCI60 human tumour cell line anticancer drug screen. Nat Rev Cancer. 2006;6: 813–823. 10.1038/nrc1951 16990858

[18] Consortium ICG. International network of cancer genome projects. Nature. 2010;464: 993–998. 10.1038/nature08987 20393554 PMC2902243

[19] Network CGA. Comprehensive molecular portraits of human breast tumours. Nature. 2012;490: 61–70. 10.1038/nature11412 23000897 PMC3465532

[20] Yang W, Soares J, Greninger P, Edelman EJ, Lightfoot H, Forbes S, et al Genomics of drug sensitivity in cancer (GDSC): a resource for therapeutic biomarker discovery in cancer cells. Nucleic Acids Res. 2013;41: D955–D961. 10.1093/nar/gks1111 23180760 PMC3531057

[21] Naulaerts S, Dang CC, Ballester PJ. Precision and recall oncology: combining multiple gene mutations for improved identification of drug-sensitive tumours. Oncotarget. 2017;8: 97025–97040. 10.18632/oncotarget.20923 29228590 PMC5722542

[22] Chang Y, Park H, Yang H-J, Lee S, Lee K-Y, Kim TS, et al Cancer Drug Response Profile scan (CDRscan): a deep learning model that predicts drug effectiveness from cancer genomic signature. Sci Rep. 2018;8: 8857 10.1038/s41598-018-27214-6 29891981 PMC5996063

[23] Ammad-ud-din M, Georgii E, Gönen M, Laitinen T, Kallioniemi O, Wennerberg K, et al Integrative and personalized QSAR analysis in cancer by kernelized Bayesian matrix factorization. J Chem Inf Model. 2014;54: 2347–2359. 10.1021/ci500152b 25046554

[24] Lee S-I, Celik S, Logsdon BA, Lundberg SM, Martins TJ, Oehler VG, et al A machine learning approach to integrate big data for precision medicine in acute myeloid leukemia. Nature Communications. 2018;9: 42 10.1038/s41467-017-02465-5 29298978 PMC5752671

[25] Camacho DM, Collins KM, Powers RK, Costello JC, Collins JJ. Next-generation machine learning for biological networks. Cell. 2018;173: 1562–1565. 10.1016/j.cell.2018.05.056 29887378

[26] Ali M, Aittokallio T. Machine learning and feature selection for drug response prediction in precision oncology applications. Biophys Rev. 2019;11: 31–39. 10.1007/s12551-018-0446-z 30097794 PMC6381361

[27] Breiman L. Random forests. Mach Learn. 2001;45: 5–32.

[28] Qi Y. Ensemble Machine Learning. Boston, MA: Springer; 2012.

[29] Nayak DR, Dash R, Majhi B. Brain MR image classification using two-dimensional discrete wavelet transform and AdaBoost with random forests. Neurocomputing. 2016;177: 232–247.

[30] Wan Q, Pal R. An ensemble based top performing approach for NCI-DREAM drug sensitivity prediction challenge. PLoS ONE. 2014;9: e101183 10.1371/journal.pone.0101183 24978814 PMC4076307

[31] Riddick G, Song H, Ahn S, Walling J, Borges-Rivera D, Zhang W, et al Predicting in vitro drug sensitivity using random forests. Bioinformatics. 2011;27: 220–224. 10.1093/bioinformatics/btq628 21134890 PMC3018816

[32] Haider S, Rahman R, Ghosh S, Pal R. A copula based approach for design of multivariate random forests for drug sensitivity prediction. PLoS ONE. 2015;10: e0144490 10.1371/journal.pone.0144490 26658256 PMC4684346

[33] Hejase HA, Chan C. Improving drug sensitivity prediction using different types of data. CPT Pharm Syst Pharmacol. 2015;4: 98–105. 10.1002/psp4.2 PMC4360670 26225231

[34] Souglakos J, Philips J, Wang R, Marwah S, Silver M, Tzardi M, et al Prognostic and predictive value of common mutations for treatment response and survival in patients with metastatic colorectal cancer. Brit J Cancer. 2009;101: 465–472. 10.1038/sj.bjc.6605164 19603024 PMC2720232

[35] Lièvre A, Blons H, Laurent-Puig P. Oncogenic mutations as predictive factors in colorectal cancer. Oncogene. 2010;29: 3033–3043. 10.1038/onc.2010.89 20383189

[36] Bresler SC, Weiser DA, Huwe PJ, Park JH, Krytska K, Ryles H, et al ALK mutations confer differential oncogenic activation and sensitivity to ALK inhibition therapy in neuroblastoma. Cancer Cell. 2014;26: 682–694. 10.1016/j.ccell.2014.09.019 25517749 PMC4269829

[37] Martins MM, Zhou AY, Corella A, Horiuchi D, Yau C, Rakhshandehroo T, et al Linking tumor mutations to drug responses via a quantitative chemical-genetic interaction map. Cancer Discov. 2014;5: 154–167. 10.1158/2159-8290.CD-14-0552 25501949 PMC4407699

[38] Gütlein M, Karwath A, Kramer S. CheS-Mapper–chemical space mapping and visualization in 3D. J Cheminformatics. 2012;4: 7. 10.1186/1758-2946-4-7 PMC3331825 22424447

[39] Willighagen EL, Mayfield JW, Alvarsson J, Berg A, Carlsson L, Jeliazkova N, et al The Chemistry Development Kit (CDK) v2.0: atom typing, depiction, molecular formulas, and substructure searching. J Cheminformatics. 2017;9: 33. 10.1186/s13321-017-0220-4 PMC5461230 29086040

[40] Rogers D, Hahn M. Extended-connectivity fingerprints. J Chem Inf Model. 2010;50: 742–754. 10.1021/ci100050t 20426451

[41] Wright MN, Ziegler A. ranger: a fast implementation of random forests for high dimensional data in C++ and R. J Stat Softw. 2017;77: 1–17.

[42] Goldstein BA, Polley EC, Briggs F. Random forests for genetic association studies. Stat Appl Genet Mol Biol. 2011;10: 32 10.2202/1544-6115.1691 22889876 PMC3154091

[43] Cohen J. A coefficient of agreement for nominal scales. Educ Psychol Meas. 1960;20: 37–46.

[44] Rücker C, Rücker G, Meringer M. y-Randomization and its variants in QSPR/QSAR. J Chem Inf Model. 2007;47: 2345–2357. 10.1021/ci700157b 17880194

[45] Arisholm E, Briand LC, Johannessen EB. A systematic and comprehensive investigation of methods to build and evaluate fault prediction models. J Syst Software. 2010;83: 2–17.

[46] Chawla NV, Bowyer KW, Hall LO, Kegelmeyer WP. SMOTE: synthetic minority over-sampling technique. J Artif Intell Res. 2002;16: 321–357.

[47] Tibshirani R. Regression shrinkage and selection via lasso. J Roy Stat Soc B. 1996;58: 267–288.

[48] Breiman L, Friedman JH, Olshen RA, Stone CJ. Classification and Regression Trees. Wadsworth; 1984.

[49] De Ferrari L, Aitken S, van Hemert J, Goryanin I. EnzML: multi-label prediction of enzyme classes using InterPro signatures. BMC Bioinformatics. 2012;13: 61 10.1186/1471-2105-13-61 22533924 PMC3483700

[50] Sherwood B, Zhou A, Weintraub S, Wang L. Using quantile regression to create baseline norms for neuropsychological tests. Alzheimers Dement. 2016;2: 12–18. 10.1016/j.dadm.2015.11.005 PMC4879644 27239531

[51] Hermeking H. The MYC oncogene as a drug target. Curr Cancer Drug Tar. 2003;3: 163–175. 10.2174/1568009033481949 12769686

[52] Solit DB, Garraway LA, Pratilas CA, Sawai A, Getz G, Basso A, et al BRAF mutation predicts sensitivity to MEK inhibition. Nature. 2006;439: 358–362. 10.1038/nature04304 16273091 PMC3306236

[53] Sterne J, White IR, Carlin JB, Spratt M, Royston P, Kenward MG, et al Multiple imputation for missing data in epidemiological and clinical research: potential and pitfalls. BMJ. 2009;338: b2393 10.1136/bmj.b2393 19564179 PMC2714692

[54] Menden MP, Iorio F, Garnett M, McDermott U, Benes CH, Ballester PJ, et al Machine learning prediction of cancer cell sensitivity to drugs based on genomic and chemical properties. PLoS ONE. 2013;8: e61318 10.1371/journal.pone.0061318 23646105 PMC3640019

[55] Cortes-Ciriano I, Mervin LH, Bender A. Current trends in drug sensitivity prediction. Curr Pharm Des. 2016;22: 6918–6927. 10.2174/1381612822666161026154430 27784247

[56] Cichonska A, Ravikumar B, Parri E, Timonen S, Pahikkala T, Airola A, et al Computational-experimental approach to drug-target interaction mapping: a case study on kinase inhibitors. PLoS Comput Biol. 2017;13: e1005678 10.1371/journal.pcbi.1005678 28787438 PMC5560747

