# Supplementary figures and images for: Predicting drug activity against cancer cells by random forest models based on minimal genomic information and chemical properties
Source: PLoS One. 2019 Jul 11;14(7):e0219774. doi: 10.1371/journal.pone.0219774 (PMC6622537; doi:10.1371/journal.pone.0219774)

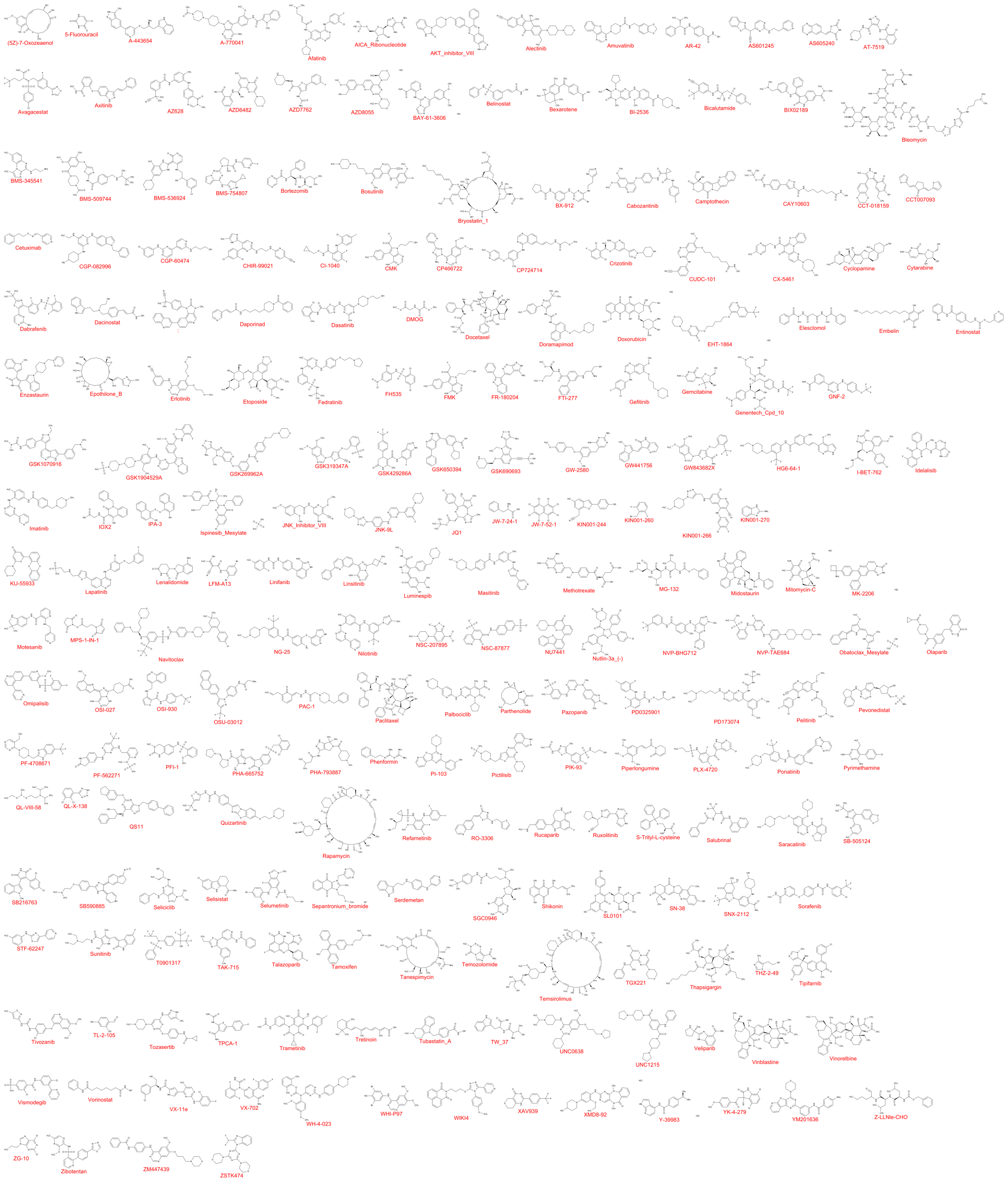

Supplement: S1 Fig — (TIF) [file pone.0219774.s001.tif]

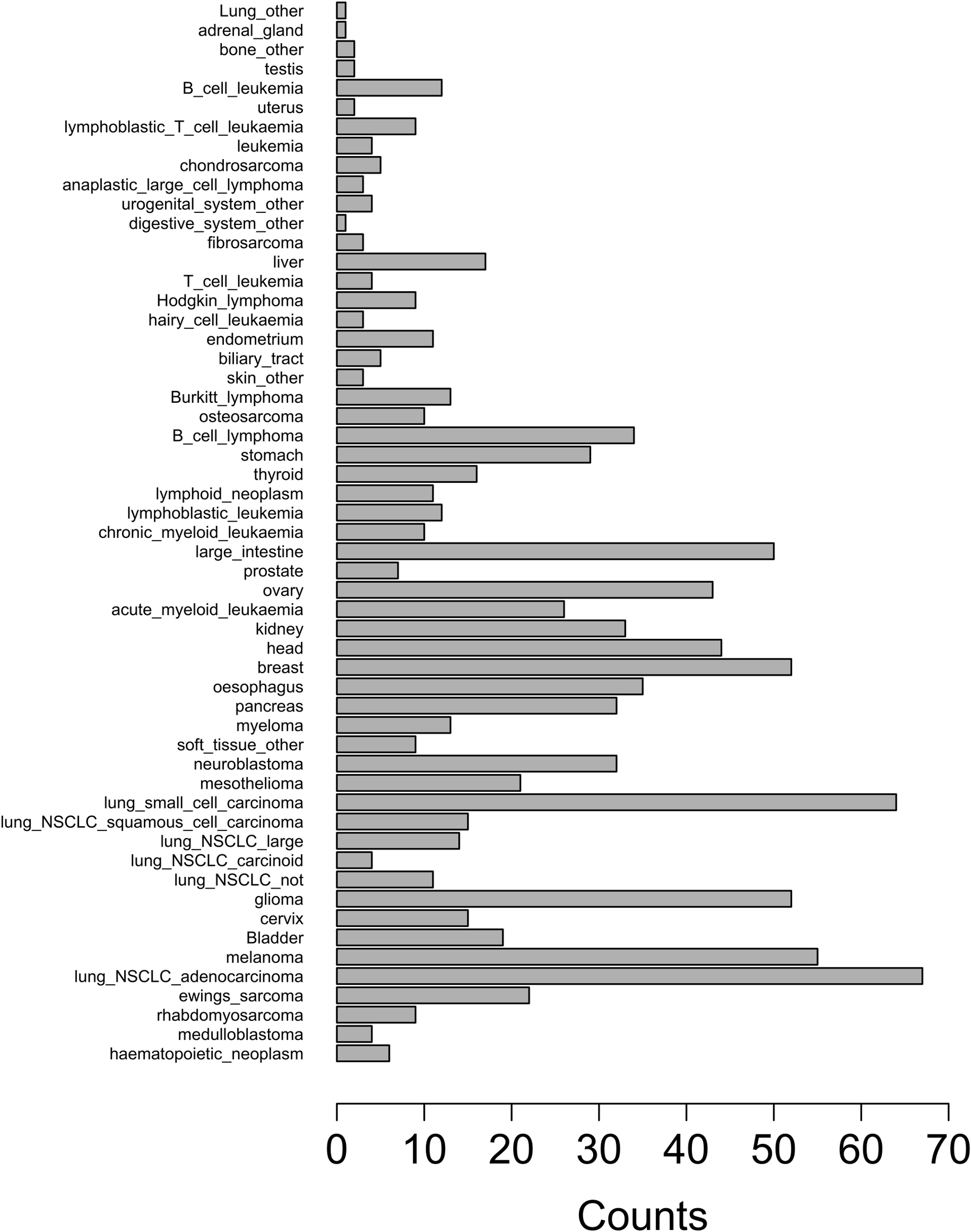

Supplement: S2 Fig — (TIF) [file pone.0219774.s002.tif]
