# Supplementary material for: Predicting drug activity against cancer cells by random forest models based on minimal genomic information and chemical properties
Source: PLoS One. 2019 Jul 11;14(7):e0219774. doi: 10.1371/journal.pone.0219774 (PMC6622537; doi:10.1371/journal.pone.0219774)
Supplement: S1 Table — (DOCX) [file pone.0219774.s003.docx]

**S1 Table.** 145 oncogenes selected from the GDSC data set as predictors of drug activity.

| *ABL1* | *CDK6* | *FLT3* | *MPL* | *REL* |
| --- | --- | --- | --- | --- |
| *ABL2* | *CDKN2C* | *FOXP1* | *MSH2* | *RET* |
| *AKT1* | *CDX2* | *FUS* | *MYB* | *ROS1* |
| *AKT2* | *CEBPA* | *GOLGA5* | *MYC* | *RUNX1* |
| *APC* | *CHEK2* | *GOPC* | *MYCL1* | *SDHB* |
| *ARHGEF12* | *CREB1* | *GPC3* | *MYCN* | *SDHD* |
| *ATF1* | *CREBBP* | *HMGA1* | *NCOA4* | *SMARCA4* |
| *ATM* | *CTNNB1* | *HMGA2* | *NF1* | *SMARCB1* |
| *BCL11A* | *CYLD* | *HRAS* | *NF2* | *SMO* |
| *BCL11B* | *DDB2* | *IDH1* | *NFKB2* | *SOCS1* |
| *BCL2* | *DDIT3* | *IL2* | *NOTCH1* | *SS18* |
| *BCL3* | *DDX5* | *IRF4* | *NPM1* | *STK11* |
| *BCL6* | *DDX6* | *JAK2* | *NR4A3* | *SUFU* |
| *BCR* | *DEK* | *JUN* | *NRAS* | *SUZ12* |
| *BLM* | *EGFR* | *KIT* | *NTRK1* | *SYK* |
| *BMPR1A* | *ELK4* | *KRAS* | *NUP214* | *TCF3* |
| *BRAF* | *ERBB2* | *LCK* | *NUP98* | *TCL1A* |
| *BRCA1* | *ETV4* | *LMO2* | *PALB2* | *TET2* |
| *BRCA2* | *ETV6* | *MAF* | *PAX8* | *TFG* |
| *CARD11* | *EVI1* | *MAFB* | *PDGFB* | *TLX1* |
| *CARS* | *EWSR1* | *MAML2* | *PIK3CA* | *TNFAIP3* |
| *CBFA2T3* | *EXT1* | *MAP2K4* | *PIM1* | *TP53* |
| *CBLB* | *EXT2* | *MDM2* | *PLAG1* | *TPR* |
| *CBLC* | *FBXW7* | *MDM4* | *PML* | *TSC1* |
| *CCND1* | *FEV* | *MEN1* | *PPARG* | *TSC2* |
| *CCND2* | *FGFR1* | *MET* | *PTEN* | *USP6* |
| *CCND3* | *FGFR1OP* | *MITF* | *PTPN11* | *VHL* |
| *CDH1* | *FGFR2* | *MLH1* | *RAF1* | *WRN* |
| *CDH11* | *FH* | *MLL* | *RB1* | *WT1* |
