# Supplementary material for: Predicting drug activity against cancer cells by random forest models based on minimal genomic information and chemical properties
Source: PLoS One. 2019 Jul 11;14(7):e0219774. doi: 10.1371/journal.pone.0219774 (PMC6622537; doi:10.1371/journal.pone.0219774)
Supplement: S2 Table — (DOCX) [file pone.0219774.s004.docx]

**S2 Table. Performance metrics for binary classification models generated by alternative machine learning algorithms.**

Support vector machine, single-layer neural network, and multi-layer deep learning network models were trained at IC_50_ cutoff values of 0.1 µM, 1 µM, and 10 µM, using 145 oncogene mutation statuses among the set of predictors. Reported errors are calculated as standard deviations from 5-fold cross-validation.

| **IC_50_ Cutoff** | **0.1 µM** | **1 µM** | **10 µM** |
| --- | --- | --- | --- |
| **Support Vector Machine** |  |  |  |
| Accuracy (%) | 93 ± 2 | 87 ± 1 | 82 ± 1 |
| Sensitivity (%) | 88 ± 4 | 87 ± 2 | 80 ± 1 |
| Specificity (%) | 94 ± 2 | 87 ± 1 | 83 ± 1 |
| False positive rate (%) | 6 ± 2 | 13 ± 1 | 17 ± 1 |
| Negative predictive value (%) | 98 ± 1 | 97 ± 1 | 82 ± 1 |
| Cohen’s kappa statistic (*κ*) | 0.86 ± 0.04 | 0.74 ± 0.02 | 0.64 ± 0.02 |
| **Single-Layer Artificial Neural Network** |  |  |  |
| Accuracy (%) | 92 ± 2 | 84 ± 1 | 77 ± 1 |
| Sensitivity (%) | 96 ± 3 | 90 ± 1 | 87 ± 1 |
| Specificity (%) | 92 ± 3 | 82 ± 1 | 68 ± 1 |
| False positive rate (%) | 8 ± 2 | 18 ± 1 | 32 ± 1 |
| Negative predictive value (%) | 99 ± 1 | 97 ± 1 | 85 ± 1 |
| Cohen’s kappa statistic (*κ*) | 0.38 ± 0.03 | 0.60 ± 0.01 | 0.54 ± 0.02 |
| **Multi-Layer Deep-Learning Network (2 Hidden Layers)** |  |  |  |
| Accuracy (%) | 94 ± 2 | 83 ± 1 | 77 ± 1 |
| Sensitivity (%) | 94 ± 3 | 90 ± 1 | 88 ± 1 |
| Specificity (%) | 94 ± 2 | 81 ± 2 | 68 ± 1 |
| False positive rate (%) | 6 ± 2 | 19 ± 2 | 32 ± 1 |
| Negative predictive value (%) | 99 ± 1 | 97 ± 1 | 86 ± 1 |
| Cohen’s kappa statistic (*κ*) | 0.44 ± 0.03 | 0.59 ± 0.02 | 0.56 ± 0.01 |
